# Recent Updates on the Functional Impact of Kahweol and Cafestol on Cancer

**DOI:** 10.3390/molecules27217332

**Published:** 2022-10-28

**Authors:** Salma Eldesouki, Rama Qadri, Rashid Abu Helwa, Hiba Barqawi, Yasser Bustanji, Eman Abu-Gharbieh, Waseem El-Huneidi

**Affiliations:** 1 College of Medicine, University of Sharjah, Sharjah 27272, United Arab Emirates; 2 Department of Clinical Sciences, College of Medicine, University of Sharjah, Sharjah 27272, United Arab Emirates; 3 Sharjah Institute for Medical Research, University of Sharjah, Sharjah 27272, United Arab Emirates; 4 Department of Basic Medical Sciences, College of Medicine, University of Sharjah, Sharjah 27272, United Arab Emirates; 5 Department of Biopharmaceutics and Clinical Pharmacy, School of Pharmacy, The University of Jordan, Amman 11942, Jordan

**Keywords:** diterpenes, anti-proliferative, cytotoxic, coffee, anticancer, cancer treatment

## Abstract

Kahweol and cafestol are two diterpenes extracted from *Coffea arabica* beans that have distinct biological activities. Recent research describes their potential activities, which include anti-inflammatory, anti-diabetic, and anti-cancer properties, among others. The two diterpenes have been shown to have anticancer effects in various in vitro and in vivo cancer models. This review aims to shed light on the recent developments regarding the potential effects of kahweol and cafestol on various cancers. A systematic literature search through Google Scholar and PubMed was performed between February and May 2022 to collect updates about the potential effects of cafestol and kahweol on different cancers in in vitro and in vivo models. The search terms “Kahweol and Cancer” and “Cafestol and Cancer” were used in this literature review as keywords; the findings demonstrated that kahweol and cafestol exhibit diverse effects on different cancers in in vitro and in vivo models, showing pro-apoptotic, cytotoxic, anti-proliferative, and anti-migratory properties. In conclusion, the diterpenes kahweol and cafestol display significant anticancer effects, while remarkably unaffecting normal cells. Our results show that both kahweol and cafestol exert their actions on various cancers via inducing apoptosis and inhibiting cell growth. Additionally, kahweol acts by inhibiting cell migration.

## 1. Introduction

Coffee is one of the most widely consumed beverages in the world. This may be attributed to its desirable stimulatory effect and distinctive taste. Fortunately, coffee’s constituents were found to have protective functions against many diseases, including type 2 diabetes mellitus, liver cirrhosis, and neurodegenerative disorders [1,2,3,4,5,6]. The physiologically active components of coffee that play this biological role include caffeine, diterpenes, chlorogenic acids, and melanoidins; their biological properties make them a hot topic to investigate.

**Figure 1:**
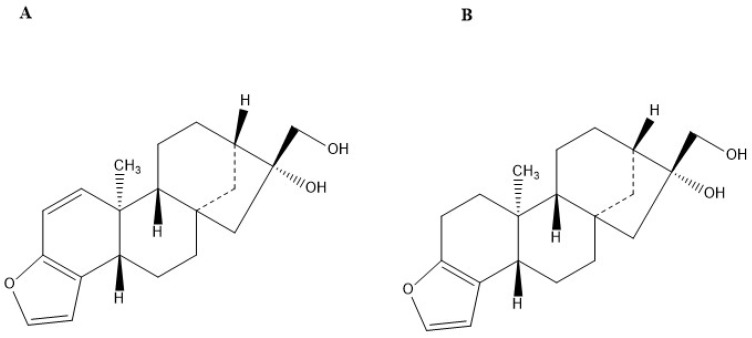
Chemical structure of (**A**) Kahweol and (**B**) Cafestol, created by ChemDraw.

The diterpenes kahweol and cafestol are fat-soluble compounds derived from Coffee arabica beans [7]. They are structural analogues, with the difference of an additional double bond in the kahweol molecule (Figure 1) [8]. Extraction of kahweol and cafestol can be performed through direct hot saponification (DHS), direct cold saponification (DCS), and Bligh and Dyer (BD) or Soxhlet (SO) extraction followed by saponification [9]. DHS was reported to be quicker, more efficient, and more economical than the other techniques for extracting the diterpenes from coffee. Purification can be performed using a flash chromatography column (FCC) packed with flash silica gel, with hexane/ethyl acetate as the mobile phase [10]. The collected fractions can be further identified by high-performance liquid chromatography with diode array and mass spectrometry detectors (HPLC–DAD–MS/MS) [9]. This method provides a simple characterization of kahweol and cafestol. In addition, it distinguishes the two molecules by their molecular mass.

In unfiltered coffee, such as Turkish coffee or espresso, kahweol and cafestol are found in relatively higher concentrations in comparison to filtered and instant coffee. A study performed on healthy ileostomy volunteers showed that, upon coffee consumption, gastric juices break down nearly 30% of kahweol and cafestol, and 64–70% of cafestol and 70–73% of kahweol are absorbed in the small intestine, with minute amounts of glucuronidated or sulphated conjugate forms being excreted in the urine [11]. As only about 1% or less of conjugate cafestol and kahweol is excreted in the urine, the two diterpenes are likely metabolised further in the body, and there is evidence that cafestol circulates in the liver and gastrointestinal tract, exerting its effects [12].

Kahweol and cafestol’s role in raising serum lipids is well-documented. The long-term ingestion of unfiltered coffee has been linked to increased plasma levels of triacylglycerol and low-density lipoprotein (LDL) cholesterol in human subjects, and cafestol and kahweol are the primary coffee constituents responsible for this effect, with cafestol exerting a more potent effect than kahweol [13]. Some in vitro studies showed that cafestol suppressed LDL receptor activity by decreasing its ability to bind, uptake, and degrade LDL [14]. However, LDL levels were also found to be raised in mice with LDL receptor knockdown following cafestol administration [15]. Cafestol suppresses enzymes involved in bile acid synthesis (sterol 27-hydroxylase and cholesterol 7 alpha-hydroxylase), providing an alternate explanation for the rise in cholesterol levels [15].

Emerging evidence has shown that kahweol and cafestol target several pathways to prevent disease. Their anti-inflammatory effect is prominently displayed by their ability to regulate several inflammatory mediators, such as ICAM1, MCP1, and IL-8, which are involved in cardiovascular disease progression [16,17]. In addition, kahweol and cafestol inhibit osteoclast differentiation and bone resorption, while promoting osteoblast differentiation, thus ameliorating degenerative bone diseases [18,19]. Of the many roles the diterpenes play, their anti-carcinogenic effect is perhaps the most significant due to the poor prognosis of many cancers.

The chemopreventive function of diterpenes can be seen in their ability to induce phase 2 detoxifying enzymes and anti-oxidant proteins, which prevents early mutagenic events. Their pro-apoptotic characteristic also contributes to their anti-tumorigenic effects by downregulating anti-apoptotic proteins, such as Bcl-2, Bcl-xL, mcl1, cFLIP, decreasing the release of cytochrome c and activating caspase enzymes [20]. In addition, diterpenes are also inhibitors of angiogenesis, as their introduction to HMVECs (Human Microvascular Endothelial Cells) inhibits cell proliferation and migration [21].

This is the first review focusing on the various effects exerted by kahweol and cafestol on different in vitro and in vivo cancer models and the pathways involved in the eradication of such cancers. The current literature primarily focuses on the general bioactivity and pharmacological effects of these two diterpenes, including their anti-inflammatory, anti-diabetic and anti-osteoclastogenesis activity, with only a cursory description of their anticancer effects [8]. Thus, this review aims to discuss, in detail, the tumour-suppressive role both kahweol and cafestol play in various cancer, through apoptosis, inhibition of proliferation and migration, or induction of cytotoxicity. The biochemical pathways through which these compounds exert their effects on multiple cancers are outlined, and their potential function in cancer treatment is explored.

## 2. Kahweol and Cafestol Effects on Several Cancer Cell Lines

Kahweol and cafestol exert anticancer effects on several cancer cell lines. The pathways through which they exhibit their properties are discussed in this section. Table 1 and Table 2 summarise the findings.

### 2.1. Lung Cancer

Kahweol and cafestol were tested in vitro against different lung cancer cell lines, and kahweol-induced apoptosis was observed in all cell lines. With regard to mesothelioma, MSTO-211H cells and H28 cells were examined [7]. The apoptosis of cancerous cells was activated by the upregulation of Bax, alongside the downregulation of Bcl-xL by kahweol and the cleavage of Bid, caspase-3, and PARP by cafestol. Kahweol’s effects were also tested on the lung adenocarcinoma cell line A549, demonstrating DNA fragmentation effects and apoptosis, a decrease in STAT3 expression, as well as an increase in caspase-3 cleavage [22]. 

Finally, non-small cell lung cancer was also tested, specifically, the cell lines NCI-H358 and NCI-H1299 [23]. Apoptosis induced by kahweol was achieved, overall, through a very similar molecular targeting that seems to be common between mesotheliomas, lung adenocarcinomas, and non-small cell lung cancers and that involves an increase in the cleavage of both PARP and caspase-3, eventually leading to apoptosis.

### 2.2. Oral Squamous Cancer

The oral squamous cancer cell lines HN22 and HSC4 were used to help assess the effects of kahweol in an in vitro environment [24]. Suppression of the transcription factor Sp1, which helps in cell differentiation, cell growth, apoptosis, response to DNA damage, and chromatin remodelling, helped achieve cancer cell apoptosis induced by kahweol.

### 2.3. Prostate Cancer

The in vitro impact of kahweol acetate and cafestol was assessed in the human prostate cancer cell lines PC-3, DU145, and LNCaP [25]. Kahweol acetate and cafestol significantly inhibited proliferation and migration in addition to enhancing apoptosis in the cell lines. Cleaved caspase-3 and its downstream target cleaved PARP, both pro-apoptotic proteins, were upregulated in all cell lines, while the anti-apoptotic proteins STAT3, Bcl-2, and Bcl-xL were diminished. Androgen receptor (AR), a strong driver of proliferation in prostate cancer, was downregulated following the treatment with kahweol acetate and cafestol. Moreover, the levels of CCL-2 and CCL-5, in addition to those of their receptors CCR-2 and CCR-5, were decreased. 

Kahweol acetate and cafestol also exhibited anti-oncogenic activity in vivo. In a xenograft study where human cell lines DU-145 were injected in SCID mouse models, the oral intake of kahweol and cafestol significantly reduced tumour growth.

### 2.4. Breast Cancer

Kahweol has been shown to exert an anti-tumour effect on breast cancer cell lines. In vitro treatment of MDA-MB231 with kahweol resulted in the inhibition of cell proliferation along with the induction of apoptosis [26]. The levels of the pro-apoptotic proteins caspase-3/7 and 9, as well as of the haemeprotein cytochrome c, were increased by kahweol treatment. Kahweol also led to an increase in H_2_O_2_ cytotoxicity in a dose-dependent manner [26].

Kahweol’s effects on the MDA-MB231 cell line can be traced back to the increased levels of phosphorylated Akt (p-Akt) and extracellular-signal-regulated kinase (ERK), activating a signalling pathway that regulates multiple cellular processes such as proliferation and apoptosis [26]. The migratory ability of the cell line was also investigated following kahweol treatment, showing reduced levels of matrix metalloproteinase-9 (MMP-9) and urokinase-type plasminogen activator (uPA).

Kahweol treatment resulted in reduced proliferation and increased apoptosis in the HER2-overexpressing SKBR3 cell line [27]. It also led to the downregulation of HER2, a growth factor essential to proliferation. To evaluate kahweol’s effect on HER2, two molecular targets were assessed: PEA3, which is a suppressor of HER2 transcription, and AP-2, which upregulates HER2. Kahweol treatment increased the levels of PEA3 while reducing those of AP-2, confirming that the two pathways were targeted by kahweol. In addition, fatty acid synthase (FASN), normally elevated in HER2-overexpressing breast cancers, was decreased in concentration, which was attributed to kahweol’s modulation of sterol regulatory element-binding protein-1c (SREBP-1c) activity. Kahweol also led to a reduction in the levels of p-Akt and of its downstream targets mTOR and cyclin D1, which had an additional impact on the downregulation of FASN [27].

### 2.5. Colorectal Cancer

Colorectal cancer cell lines have also been shown to be susceptible to kahweol. Kahweol induced apoptosis in HCT116 cells in vitro through the overexpression of ATF3, which is mediated by CREB1, ERK1/2, and GSK3β [28]. In addition, kahweol suppressed the proliferation in vitro of HCT116 and SW480 cells, as evidenced by their reduced levels of cyclin D1, a cell cycle protein that promotes progression through the cell cycle [29]. Kahweol-mediated degradation of cyclin D1 was achieved through the phosphorylation of cyclin D1 at threonine-286 (Thr286). This effect was attenuated upon the inhibition of any of kahweol’s molecular targets ERK1/2, JNK, and GSK3β. 

Kahweol’s anti-apoptotic characteristics were also observed in the human colon adenocarcinoma HT-29 cells [30,31]. Cell death was induced by kahweol treatment in a dose-dependent manner, which was further proven by the elevation of the pro-apoptotic markers cleaved caspase-3 and PARP, as well as the reduction of the anti-apoptotic markers Bcl-2 and p-AKT. Furthermore, the levels of heat shock proteins (HSP40, HSP70, and HSP90) were diminished following kahweol treatment. HSPs are a family of molecular chaperones that prevent cell death, and their downregulation is associated with increased cytotoxicity of tumour cells.

### 2.6. Renal Carcinoma

Kahweol’s anti-tumour properties were assessed in several renal carcinoma cell lines in vitro, with a predominant focus on Caki cells. TRAIL, a natural ligand for death receptors found on cancer cells, exerted its apoptotic function on Caki cells more effectively when it was combined with kahweol [33]. Co-treatment with TRAIL and kahweol strongly activated DEVDases and reduced the levels of c-FLIP and Bcl-2, which are anti-apoptotic proteins. The JNK and p38 MAPK pathways were found to mediate these effects. Similarly, the treatment of Caki cells with melatonin and kahweol exhibited comparable findings, which included the induction of apoptosis and the activation of DEVDases [32]. The levels of the pro-apoptotic Bcl-2 protein from the p53 Upregulated Modulator of Apoptosis (PUMA) protein family were also upregulated in response to the treatment. This upregulation was achieved through the C/EBP homologous protein (CHOP), a pro-apoptotic transcription factor. Furthermore, co-treatment with kahweol and sorafenib, a tyrosine kinase inhibitor, had analogous effects on renal carcinoma cells [34]. Caspase-dependent apoptosis and downregulation of c-FLIP and Mcl-1 were induced by those compounds when tested on Caki cells as well as on other renal carcinoma cell lines, including A498 and ACHN cells.

Cafestol was also shown to exhibit its anti-carcinogenic effect in renal Caki cells. Cafestol-induced apoptosis was found to be mediated by the activation of caspase-2 and 3, the upregulation of the pro-apoptotic proteins Bim and Bax, and the downregulation of anti-apoptotic proteins, including c-FLIP, Bcl-2, Mcl-1, and Bcl-xL [31]. The apoptotic effect was also achieved by the inhibition of both STAT3 activation and the PI3K/Akt pathway.

The combined anti-tumour activity of kahweol acetate and cafestol was documented in renal Caki and ACHN cell lines [35]. In these cells, the induction of apoptosis and epithelial–mesenchymal transition upon administration of the diterpenes inhibited proliferation and migration. The inhibition of STAT3 activation and the downregulation of Bcl-2- and Bcl-xL-mediated the apoptosis was exerted by the diterpenes, alongside the upregulation of Bax. Moreover, the diterpenes inhibited Akt and ERK phosphorylation, which are both known to accelerate metastasis and tumour growth.

Finally, cafestol was tested on the Caki cells both in vitro and in vivo using BALB/c-nude mice as the cancer cells recipients [36]. Anti-cancer effects were induced via the synergistic impacts of cafestol and ABT-737, a Bcl-2 family inhibitor. The inactivation of Bcl-2 proteins (Bcl-2, Bcl-xL, and Bcl-w) helped induce apoptosis. PARP cleavage was also increased by the combined actions of cafestol and ABT-737. Mcl-1 protein was also downregulated by this combination in the in vivo setting, leading overall to a pro-apoptotic effect on Caki cells.

### 2.7. Leukaemia

The U937 leukaemia cell line was tested on in vitro to evaluate kahweol’s apoptotic effect, which was found to be mediated by the activation of caspase-3 and the downregulation of Bcl-2 [38]. The AKT and JNK pathways were also found to be associated with this effect.

Other cell lines such as NB4, K562, HL60, and KG1 were also tested in vitro using cafestol and Ara-c, which is an anti-leukemic agent that was used as a positive control [39]. Increased caspase-3 cleavage was observed in HL60 cells, which helped induce apoptosis via cafestol. Finally, cafestol also helped increase the expression of CD15 and CD11b and decrease the formation of ROS. 

Kahweol and cafestol were also shown to enhance the activity of the NK cell line KHYG-1 and its cytolytic effect on the NK-sensitive leukaemia cell line K562. The two diterpenes increased granzyme B expression in NK cells, likely through the phosphorylation of ATF-2, c-Jun, and CREB, transcription factors involved in the regulation of granzyme B. The net result was the enhancement of the cytolytic activity of NK cells in leukaemia cell lines [40].

### 2.8. Fibrosarcoma

Kahweol acetate was found to attenuate cancer formation, proliferation, and migration by the fibrosarcoma HT-1080 cell line by inhibiting MMP-9, which is usually upregulated by PMA, 12-phorbol 13-myristate acetate, a synthetic compound known for its oncogenic effect [30]. Kahweol acetate was found to act by inhibiting MMP-9, which is usually upregulated by PMA. This inhibition was achieved by the suppression of PMA-induced NF-κB activity, alongside the suppression of the Akt, p38 MAPK, and JNK1/2 signalling pathways, which were also found to activate MMP-9.

### 2.9. Hepatocellular Carcinoma

In vitro, kahweol exerted apoptotic effects as well as inhibited cell proliferation in the hepatocellular carcinoma cell lines Hep3B, SNU182, and SNU42 [37]. The Src signalling pathway, which is activated upon the phosphorylation of Src, is highly functional in HCC. The Src pathway was blocked by kahweol by inhibiting the expression of p-Akt, p-mTOR, p-p70S6K, and p-4EBP1 in Hep3B and SNU182 cells. This had a direct apoptotic and anti-proliferative effect on the cancer cells. mTOR, which has a proliferative function, was also inhibited by kahweol. Finally, STAT3 was also blocked by kahweol, which induced a pro-apoptotic effect in the HCC cell lines.

### 2.10. Head and Neck Squamous Cell Carcinoma

Upon the addition of cafestol in vitro, the cell lines of SCC25, CAL27 and FaDu were found to undergo apoptosis in a dose-dependent manner. When combined with cisplatin, cafestol displayed an antagonistic effect in all cell lines; moreover, its addition to radiation therapy showed an additive effect in the cell lines SCC25 and CAL27 [41]. Very limited studies have been performed on head and neck squamous cell carcinoma, which calls for more extensive research on the topic.

## 3. Conclusions and Future Perspectives

**Figure 2:**
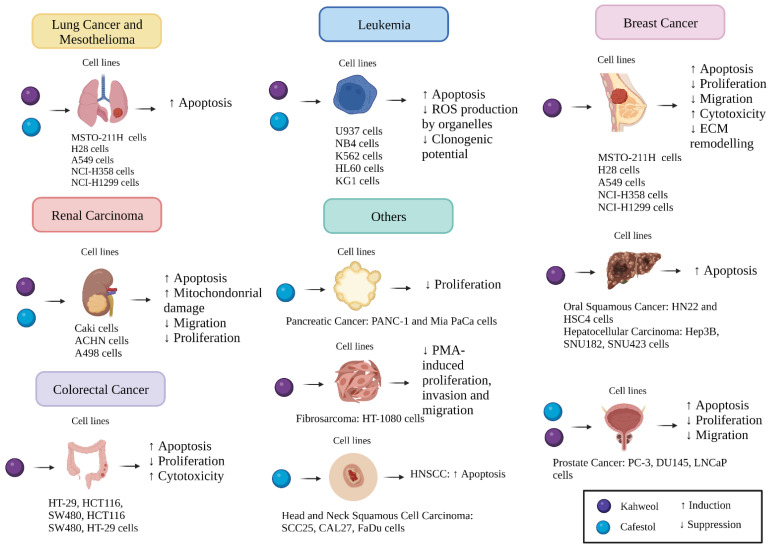
A diagram summarising the potential anti-cancer mechanisms of kahweol and cafestol in different cancer cell lines. Created by BioRender.com.

Diterpenes are fat-soluble compounds that can be derived from *Coffea arabica* beans and are known for their disease-preventing characteristics. Kahweol and cafestol, two diterpenes, were found to have a striking role in preventing the progression of several types of cancer, as discussed above. This effect is achieved mainly by inducing apoptosis, alongside cytotoxicity and mitochondrial damage, which are mediated by targeting certain downstream molecules and pathways, as summarised in Figure 2.

**Figure 3:**
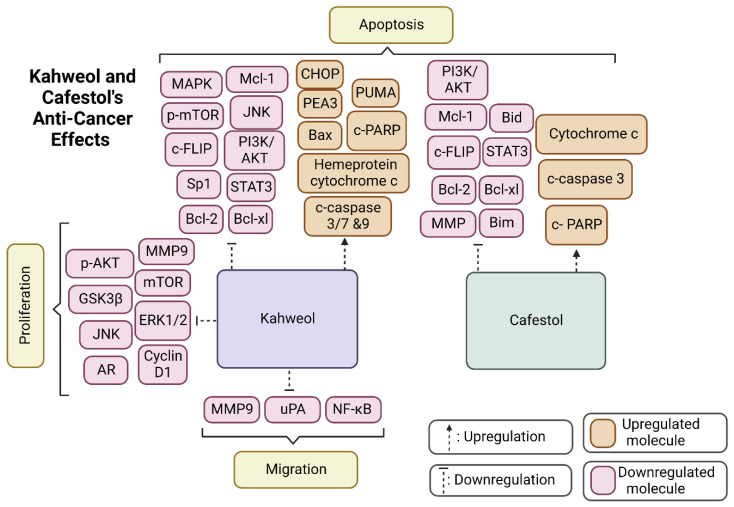
The diagram summarises kahweol and cafestol’s effects on anticancer pathways, including migration, proliferation, and apoptosis. Created by BioRender.com.

Figure 3 portrays the most common molecular targets of kahweol and cafestol, through which they exhibit their tumour-suppressive function. The two diterpenes, as depicted in the figure, either upregulate anti-cancer pathways, such as c-PARP and c-caspase, leading to the apoptosis of cancer cells, or downregulate molecules, including Bcl-2 and Bcl-xL, which also achieves an apoptotic effect.

Anticancer properties can also be exerted by other diterpenes and their derivatives. Taxanes, a well-known group of chemotherapeutic agents, are a class of diterpenes extracted from the plant genus *Taxus*, indicated for the treatment of breast, ovarian, and prostate cancers, among others [42]. Another diterpene, triptolide, has long been held as a promising anticancer agent [43]. It possesses anti-proliferative and pro-apoptotic properties, inducing cell apoptosis through the upregulation of cleaved caspase-3, cleaved caspase-9, cleaved PARP, and Bax and the downregulation of Bcl-2, in a similar way as kahweol and cafestol [44]. 

The chemotherapeutic role of both diterpenes is mainly exerted in cancer cell lines, with a possible minimal effect on normal body cells. The anticancer effects of these diterpenes are dose-dependent and time-dependent. According to the current literature, the doses of kahweol and cafestol required to exert their anticancer properties are, dependent on the type of cancer and cell line. For example, kahweol suppressed proliferation and promoted apoptosis in lung adenocarcinoma A549 cells at a dose of 10–40 μM when administered for 24 to 48 h [22]. Similarly, an in vitro study reported that 20–80 μM cafestol inhibited human umbilical vein endothelial cells (HUVEC) proliferation in a dose-dependent manner, ultimately achieving cafestol’s anti-angiogenic effects [8]. The precise impact of cafestol and kahweol on normal body cells has not been thoroughly studied and thus warrants further investigation in order to accurately determine the toxicity of these compounds.

Compared to the anticancer doses of kahweol and cafestol, their anti-inflammatory doses are lower. Both diterpenes can inhibit the production of PGE2 and NO, two inflammatory modulators, in lipopolysaccharide (LPS)-activated macrophages in a dose-dependent manner [8]. For instance, at a dose of 0.5–10 μM, kahweol and cafestol can inhibit the activation of IκB kinase (IKK), which is the main activator of the inflammatory transcription factor NF-κB, in LPS-activated macrophages [45]. Furthermore, at the small dose of 0.5 μM, kahweol can potently inhibit cyclooxygenase-2 (COX-2), which is an enzyme that increases the levels of the inflammatory modulators PGE2 and NO [45]. 

Alongside their potent anti-inflammatory roles, kahweol and cafestol’s anti-oxidant effects are also prominent. The diterpenes exhibit the ability to induce the Keap1/Nrf2/ARE signalling pathway, which is primarily responsible for protection against reactive oxygen species within the liver cells [8]. This activity, like its anti-inflammatory function, is dose-dependent. Lee et al. highlighted the diterpenes’ anti-oxidant ability by using them to treat tetrachloride-induced liver damage in mice [46]. By scavenging free radicals and limiting lipid peroxidation, the diterpenes were able to suppress liver damage, starting from a dose of 20 μM. At higher doses of 100 μM and 200 μM, liver injury was further decreased. Unfortunately, this study was conducted in 2007 [46], and no recent literature highlights any advancements regarding the diterpenes’ anti-oxidant effects, making it an interesting topic to revisit.

Diterpenes have also been utilised in the treatment of other diseases. Vitamin A, a lipid-soluble diterpene, is a dietary supplement that is essential for human health, as it has functions in vision, early growth and development, as well as higher brain functions such as learning and memory [47]. Moreover, Vitamin A is used to produce Tretinoin, an FDA-approved drug that is indicated for the treatment of acne vulgaris, psoriasis, and cutaneous warts [48]. Interestingly, it has also been shown to induce remission in patients with acute promyelocytic leukaemia. Phytol is a diterpene alcohol that, along with its derivatives, displays a multitude of biological functions, such as anxiolytic, antimicrobial, anti-inflammatory, and immune-modulating effects [49]. Vitamin K1 can be extracted from phytol and used in the treatment of clotting factor disorders [50,51].

In conclusion, this review provides sufficient evidence portraying the anticancer role of kahweol and cafestol in vitro and in vivo. Inhibition of proliferation and the induction of apoptosis are the primary mechanisms by which the two diterpenes exert their tumour-suppressive effects. The literature surrounding in vitro studies is impressive, especially in regard to renal cell carcinoma, breast cancer, leukaemia, and colorectal cancer, as the tumour-suppressive effects of kahweol and cafestol were verified in numerous studies. Other cancer cell lines, including mesothelioma, lung adenocarcinoma, non-small cell lung cancer, oral squamous cancer, prostate cancer, hepatocellular carcinoma, fibrosarcoma, and head and neck squamous cell carcinoma, were tested using the two diterpenes in no more than two in vitro studies. Prostate cancer and renal cell carcinoma cells are the only cell lines used in in vivo studies, which confirmed their potential response to kahweol and cafestol, calling for a more extensive clinical investigation. As such, the chemotherapeutic function of these diterpenes should be assessed alongside their pharmacokinetics, including their absorption, bioavailability, metabolism, and elimination, as well as their safety profile via toxicological testing. The two diterpenes have a promising potential to revolutionise the field of cancer treatment.

## 4. Methods

**Figure 4:**
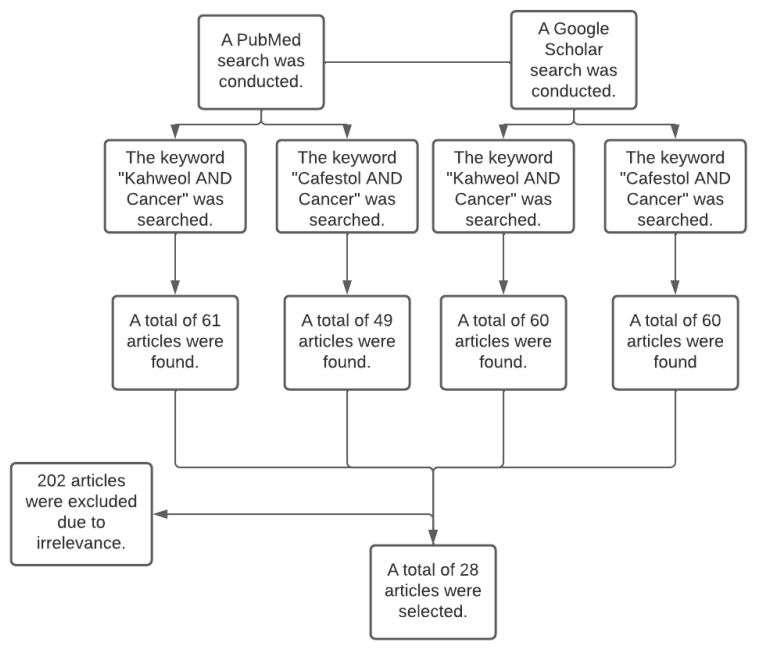
The flowchart represents the data collection process, including the used databases (PubMed and Google Scholar), the keywords, and the number of included and excluded articles.

A systematic literature search was conducted in the databases PubMed and Google Scholar between February 2022 and May 2022. The search terms were “Kahweol and Cancer” and “Cafestol and Cancer”. Each term was searched separately on both databases, and the articles whose title or abstract was found to be relevant were filtered based on the inclusion criteria. The researchers independently selected the articles that discussed the effect of kahweol and cafestol on human cancer cell lines in vivo and in vitro and discarded the articles that did not discuss the anticancer effects of the two diterpenes. Figure 4 summarises the data collection process.

## Figures and Tables

**Table 1:** In Vitro Impact of Kahweol and Cafestol on Cancer Cell Lines (↑ indicates induction, ↓ indicates suppression).

Cancer Type		Cell Line	Coffee Derivative	Molecular Target	Functional Impact	Ref.
Mesothelioma		MSTO-211H cells H28 cells	Kahweol and cafestol	↑ Bax ↓ Bcl-xl ↑ Cleavage of Bid, Caspase-3, and PARP ↓ Sp1	↑ Kahweol-induced apoptosis	[7]
Lung Adenocarcinoma		A549 cells	Kahweol	↑ Bax ↓ Bcl-2 ↓ Bcl-xl ↑ Cleavage of Caspase-3 and PARP ↓ STAT3	↑ DNA fragmentation ↑ Caspase-3-mediated apoptosis ↑ STAT3-mediated apoptosis	[22]
Non-small Cell Lung Cancer		NCI-H358 cells NCI-H1299 cells	Kahweol	↓ BTF3 ↓ ERK signalling pathway ↑ Cleavage of PARP and Caspase-3 ↑ p27 and p21 ↓ cyclin D1 ↑ Bax ↓ Bcl-2 ↓ Bcl-xl	↑ Kahweol-induced apoptosis	[23]
Oral Squamous Cancer		HN22 cells HSC4 cells	Kahweol	↓ Sp1 ↑ p27 and p21 ↓ cyclin D1, Mcl-1, and survivin ↑ Cleavage of Bid, Caspase-3, and PARP ↓ Bcl-xl ↑ Bax	↑ Kahweol-induced apoptosis	[24]
Prostate Cancer		PC-3 DU145 LNCaP	Kahweol acetate Cafestol	↑ Caspase-3 cleavage ↑ PARP cleavage ↓ Bcl-2 ↓ Bcl-xL ↓ AR ↓ CCL2-CCR2 ↓ CCL5-CCR5	↓ Proliferation ↓ Migration ↑ Apoptosis	[25]
Breast Cancer		MDA-MB231 ZR75-1 MCF-7	Kahweol	↑ Caspases-3/7, 9 ↑ Cytochrome C ↑ H2O2	↓ Proliferation ↑ Apoptosis ↑ H2O2 cytotoxicity	[26]
		MDA-MB231	Kahweol	↑ Caspases-3/7, 9 ↑ Cytochrome C ↑ p-AKT ↑ ERK ↓ MMP-9 ↓ uPA	↑ Apoptosis ↓ Migration ↓ ECM remodelling	[26]
		SKBR3MCF-10A	Kahweol	↑ PARP cleavage via ↑ caspase 3 ↓ HER2 via ↑ PEA3 and ↓ AP-2 ↓ FASN via ↓ SREBP-1c, ↓ p-Akt ↓ cyclin D1 via ↓ mTOR, ↓ GSK-3β	↑ Apoptosis ↑ Cytotoxicity ↓ Proliferation	[27]
Colorectal Cancer		HCT116 SW480 LoVo HT-29	Kahweol	↑ PARP cleavage via ↑ ATF3	↑ Apoptosis	[28]
		HCT116 SW480	Kahweol	↓ cyclin D1 via ↑ Thr286	↓ Proliferation	[29]
		HT-29	Kahweol	↑ Caspase-3 cleavage ↑ PARP cleavage ↓ Bcl-2 ↓ p-AKT ↓ HSP40, HSP70, HSP90	↑ Apoptosis ↓ Proliferation	[20,30]
		HT-29	Kahweol	↑ Caspase-3 ↑ PARP cleavage ↓ Bcl-2 ↓ p-AKT ↓ HSP-70	↑ Apoptosis ↑ Cytotoxicity	[31]
Renal Carcinoma		Caki Cells	Kahweol	↓ Bcl-2 ↓ c-FLIP ↑ Cleavage of PARP ↑ DEVDase	↑ TRAIL-mediated apoptosis	[32]
		Caki Cells	Kahweol	↑ PUMA via ↑ CHOP ↑ DEVDase	↑ p53-independent apoptosis ↑ ER stress-mediated apoptosis	[33]
		Caki cells ACHN cells A498 cells	Kahweol	↓ Mcl-1 ↓ c-FLIP	↑ Caspase-mediated apoptosis	[34]
		Caki cells	Cafestol	↓ Mcl-1 ↓ c-FLIP ↓ MMP ↑ Cytochrome C ↑ Caspase-3 ↓ Bcl-2, Bcl-xL, Mcl-1, c-FLIP ↓ PI3K/Akt pathway	↑ Mitochondrial damage ↑ Apoptosis	[20]
		Caki-1 cells ACHN cells	Kahweol acetate and cafestol	↓ Akt and ERK phosphorylation ↓ CCR2, CCR5 & CCR6↓ PD-L1	↓ Migration ↓ Proliferation ↓ Epithelial-mesenchymal transition ↑ Apoptosis	[35]
		Caki cells	Cafestol	↑ Cleavage of PARP ↑ Caspase-3 activity ↓ Mcl-1 ↑ PUMA and Bim	↑ ABT-737-mediated apoptosis	[36]
Hepatocellular Carcinoma		Hep3B cells SNU182 cells SNU423 cells	Kahweol	↑ Cleavage of PARP and caspase 3 ↓ p-Src ↓ expression of p-Akt, p-mTOR, p-p70S6K, and p-4EBP1 ↓ p-STAT3	↑ Kahweol-induced apoptosis	[37]
Leukemia	U937 cells	Kahweol	↑ Caspase 3 ↑ Cytochrome C release ↓ Bcl-2, Bcl-xL, Mcl-1, XIAP ↓ Akt pathways ↑ JNK pathways		↑ Apoptosis	[38]
	NB4, K562, HL60 and KG1	Cafestol Ara-C	↑ Caspase 3 ↑ CD11b and CD15		↑ Apoptosis ↓ ROS (Reactive Oxygen Species) production by organelles ↓ Clonogenic potential	[39]
	K562	Kawheol and cafestol	↑ Granzyme B via ↑ ATF-2, c-Jun, and CREB phosphorylation		↑ Cytolysis	[40]
Fibrosarcoma	HT-1080 cells	Kahweol acetate	↓ PMA-induced MMP-9 via ↓ NF-κB ↓ Akt/JNK1/2/p38 MAPK phosphorylation		↓ PMA-induced proliferation, invasion, and migration	[30]
Head and Neck Squamous Cell carcinoma	SCC25 CAL27 FaDu	Cafestol Cisplatin	No mention of any pathway		↑ Apoptosis	[41]

**Table 2:** In Vivo Impact of Kahweol and Cafestol on Cancer models (↑ indicates induction, ↓ indicates suppression).

Cancer Type	Animal Model	Cell Line	Kahweol/Derivative	Molecular Target	Functional Impact	Ref.
Prostate Cancer	SCID mice	DU-145	Kahweol acetate	↑ Caspase-3 cleavage ↑ PARP cleavage ↓ Bcl-2 ↓ Bcl-xL ↓ AR ↓ CCL2-CCR2 ↓ CCL5-CCR5	Inhibition of tumour growth	[25]
Renal cell carcinoma	BALB/c-nude mice	Caki cells	Cafestol	↑ Cleavage of PARP ↑ caspase-3 activity ↓ Mcl-1 ↑ PUMA and Bim	↑ABT-737-mediated apoptosis	[39]

## Data Availability

Data are contained within the article.

## Abbreviations

AP-2	Activator Protein 2
AR	Androgen Receptor
ATF3	Cyclic AMP-dependent transcription factor
BALB/c-nude mice	Bagg and Albino/c-nude mice
Bax	BCL2-Associated X Protein
Bcl-2	B-cell leukemia/lymphoma 2 protein.
Bcl-xL	B-cell lymphoma-extra large
Bid	BH3 interacting-domain death agonist
Bim	Bcl-2-like protein 11
BTF3	Basic transcription factor 3
CCL2-CCR2	Chemokine (C-C motif) ligand 2-CC chemokine receptor 2
CCL5-CCR5	Chemokine (C-C motif) ligand 5-CC chemokine receptor 5
CCR6	CC chemokine receptor 6
CD11b	Cluster of differentiation 11b
CD15	Cluster of differentiation 15
cFLIP	Cellular FLICE (FADD-like IL-1β-converting enzyme)-inhibitory protein
CHOP	C/EBP Homologous Protein
DEVDase	Caspase-3-like proteases
ECM	Extra-cellular Matrix
ER	Endoplasmic Reticulum
ERK signaling	Extracellular signal-regulated kinase
FASN	Fatty Acid Synthase
FDA	Food and Drug administration
GSK-3β	Glycogen synthase kinase-3 beta
HER2	Human epidermal growth factor receptor 2
HMVECs	Human microvascular endothelial cell-1
HSP40/70/90	Human Heat shock protein 40/70/90
ICAM1	Intercellular adhesion molecule 1
IL-8	Interleukin-8
JNK	Jun N-terminal kinase
LDL	Low-density Lipoprotein
MAPK	Mitogen-activated protein kinase
mcl1	Myeloid cell leukemia-1
MCP1	Monocyte chemoattractant protein-1
MMP-9	Matrix metallopeptidase 9
mTOR	Mammalian target of rapamycin
NK-kB	nNuclear factor kappa light chain enhancer of activated B cells
p-4EBP1	Phosphorylated eukaryotic translation initiation factor
p-AKT	Phosphorylated Protein kinase B
PARP	Poly-ADP ribose polymerase
PD-L1	Programmed death-ligand 1
PEA3	Polyoma enhancer activator 3
PI3K/Akt pathway	Phosphatidylinositol 3-kinase/Protein kinase B
PMA	Phorbol 12-myristate 13-acetate
p-mTOR	Phosphorylated mammalian target of rapamycin
p-p70S6K	Phosphorylated Ribosomal protein S6 kinase beta-1
p-Src	Phosphorilated sarcoma
p-STAT3	Phosphorylated signal transducer and activator of transcription 3
PUMA	p53-upregulated modulator of apoptosis
ROS	Reactive oxygen species
SCID mice	Severe combined immunodeficiency mice
Sp1	Specificity protein 1
SREBP-1c	Sterol regulatory element-binding protein-1
STAT3	Signal transducer and activator of transcription 3
Thr286	Threonine 286
TRAIL-Mediated apoptosis	Tumor necrosis factor-related apoptosis-inducing ligand
uPA	Urokinase-type plasminogen activator (uPA)
XIAP	X-linked inhibitor of apoptosis protein
